# Intradural Thoracic Arachnoid Cyst Fenestration for Spinal Cord Compression: A Case Illustration and Video Demonstration

**DOI:** 10.7759/cureus.6572

**Published:** 2020-01-05

**Authors:** James Ebot, Ricardo Domingo, Henry Ruiz Garcia, Selby Chen

**Affiliations:** 1 Neurosurgery, Mayo Clinic, Jacksonville, USA; 2 Radiation Oncology and Neurosurgery, Mayo Clinic, Jacksonville, USA

**Keywords:** arachnoid cyst fenestration, spinal cord compression, thoracic spine, ultrasound

## Abstract

Spinal intradural arachnoid cysts are rare, benign intradural lesions of the spinal cord that can arise as a primary lesion or secondary due to inflammatory processes. Symptoms can range from an asymptomatic incidental finding to progressive myelopathy, with paresthesia and neuropathic pain. We present the case of an 80-year-old female with a longstanding history of back pain, urinary incontinence, difficulty ambulating and frequent falls, with rapid progression of her symptoms prior to presentation. Physical examination revealed lower extremity weakness, decreased sensation and increased deep tendon reflexes. Thoracic spine MRI showed an extra-axial cystic lesion extending from T4 to T10, causing severe compression of the spinal cord. We performed two separate thoracic laminectomies at T4-T5 and at T9-T10, with microsurgical fenestration of the dorsal arachnoid cyst performed under continuous intraoperative neurophysiologic monitoring. Intraoperative fluoroscopy and ultrasound were used for localization purposes. The patient was discharged on postoperative day 6 to an inpatient rehabilitation facility with no neurological complications. She presented a month later with significant improvement in ambulation and lower extremity strength.

## Introduction

Spinal arachnoid cysts are rare, usually benign intradural extramedullary lesions of the spinal cord that can arise as a primary lesion, or secondary due to post-traumatic or postinfectious causes [1]. Symptoms can range from an asymptomatic incidental finding to progressive myelopathy, with paresthesia and neuropathic pain. Spinal arachnoid cysts are usually diagnosed either as an incidental finding or in patients with a long history of back pain with or without myelopathy who finally undergo neuroimaging [2]. On magnetic resonance imaging (MRI), they are usually dorsal lesions with an intensity that is similar to cerebrospinal fluid (CSF) [3]. It occurs most commonly in the thoracic spine, followed by the cervical spine and least in the lumbar spine [4]. Treatment options range from conservative management for incidentally discovered lesions with no neurological deficits to surgical excision or fenestration for patients presenting with symptomatic lesions.

## Case presentation

The patient is an 80-year-old female with a past medical history of hypertension, hyperlipidemia, gastro-esophageal reflux disease and chronic obstructive pulmonary disease who presented with a longstanding history of back pain, urinary incontinence, difficulty ambulating and frequent falls, with rapid progression of her symptoms prior to presentation. Her physical examination revealed lower extremity weakness, decreased sensation and increased deep tendon reflexes. Her thoracic spine MRI showed an intradural, extramedullary cystic lesion extending from T4 to T10, causing severe compression of the spinal cord. We performed two separate thoracic laminectomies at T4-T5 and at T9-T10, with microsurgical fenestration of the dorsal arachnoid cyst performed under continuous intraoperative neurophysiologic monitoring. Intraoperative fluoroscopy and ultrasound were used for localization purposes (Video 1). The patient did really well and was discharged on postoperative day 6 to an inpatient rehabilitation facility with no neurological complications. At her one-month follow-up visit, her lower extremity strength had improved and she was ambulating much better.

**Video 1:** Thoracic Arachnoid Cyst Fenestration for Spinal Cord Compression Step-by-step techniques for performing a thoracic arachnoid cyst fenestration for a long lesion causing spinal cord compression. Yo – year old, PMH – past medical history, HTN – hypertension, COPD – chronic obstructive pulmonary disease, GERD – gastro-esophageal reflux disease, R – right,  L – left, MRI – magnetic resonance image, POD – postoperative day.

## Discussion

We describe a case of progressive back pain, lower extremity weakness and incontinence caused by a long segment dorsal arachnoid cyst of the thoracic spine. Traditionally, the surgical approach will entail one long incision spanning the T4-T10 spinal segments in other to gain access to the entire lesion [3]. This will require a large incision, resulting in more blood loss intraoperatively with a large area of wound exposed to potential infections. It will also require performing a laminectomy over many levels to have adequate exposure to the spine, thereby disrupting the bony and ligamentous complex of the posterior column, which can cause some instability. Our goal of treatment was to decompress the spinal cord and relieve the compression via fenestration of the arachnoid cyst, but also ensure that there is good communication of CSF between the opposite ends of the lesion post fenestration. We elected to perform two small incisions, one at the beginning and the other at the end of the lesion, instead of one large incision. Using an ultrasound, we were able to directly visualize the cyst and CSF flow across the area. After opening the dura on both ends of the cyst, we were able to ensure continuous passage of a catheter from one end of the incision to the other. At the end of the fenestration, we were again able to confirm disappearance of the cyst using ultrasound, with improved flow of CSF across the area. We believe this approach was ideal because it reduced operative time significantly, subjecting the patient to significantly less general anesthesia, which led to a much faster recovery and discharge from the hospital. Also, performing two small incisions instead of one long continuous incision reduced the amount of spinal tissue exposed to the surgical field, thereby decreasing the chances of potential damage to the spinal cord.

## Conclusions

Spinal intradural arachnoid cysts are rare, usually benign intradural lesions of the spinal cord that can be found incidentally, or present with worsening neurological deficits. Management usually range from observation with follow-up imaging for asymptomatic patients to surgical decompression or fenestration for patients who present with neurological deficits. For posterior spinal arachnoid cyst spanning a long spinal segment, a less invasive approach using two small incisions at both ends of the lesion to fenestrate the cyst can offer a good decompression and also minimal blood loss, shorter operative time, quicker recovery and early discharge from the hospital.
